# The last two decades of life course epidemiology, and its relevance for research on ageing

**DOI:** 10.1093/ije/dyw096

**Published:** 2016-10-06

**Authors:** Yoav Ben-Shlomo, Rachel Cooper, Diana Kuh

**Affiliations:** 1 School of Social and Community Medicine, University of Bristol, Bristol, UK; 2 MRC Unit for Lifelong Health and Ageing, University College London, London, UK

## Introduction

The term ‘life course epidemiology’ was coined in 1997 with the publication of the first edition of *A Life Course Approach to Chronic Disease Epidemiology*.^1^ This book reviewed the pre-adult risk factors for cardiometabolic and respiratory disease, the catalyst being the imaginative research on the fetal origins of adult disease being driven forward at that time by Professor David Barker. We defined life course epidemiology as ‘the study of long-term biological, behavioural and psychosocial processes that link adult health and disease risk to physical or social exposures acting during gestation, childhood, adolescence, earlier in adult life or across generations’.^1^ Although our definition of life course epidemiology has stood the test of time, the field has evolved and there have been conceptual developments, methodological innovations which facilitate efforts to test these concepts, and an increasing corpus of empirical research demonstrating how factors from earlier life are associated with later life health and disease, as well as the pathways and biological mechanisms that may be involved. These developments have generated further ideas and challenges to life course models in an iterative process. As the theme of this special issue suggests, one important development has been the gradual shift of research focus from clinical disease endpoints to multi-faceted traits and longitudinal trajectories of functional phenotypes that can be assessed well before any clinical threshold is reached. This has naturally led on to the application of a life course epidemiological approach to ageing. The purpose of this overview is therefore to assess the development and current state of the field of life course epidemiology, including its recent application to the study of ageing as the focus of this special issue.

## Developments in the field of life course epidemiology

Life course epidemiology is now recognized as a field of research in its own right and was added to the fifth edition of the *Dictionary of Epidemiology*.^2^ The original life course book was to become the first in a series on a life course approach to adult health,^3–8^ as well as a journal glossary^9^ and chapters in a range of public health, epidemiology and other academic textbooks.^10–14^ The original *International Journal of Epidemiology* editorial on life course epidemiology, published in 2002,^15^ is the fifth most highly cited article in the journal’s history (search run on Web of Science core collection 19 February 2016).

In order to chart key developments in the field, we have drawn on our own experiences and the results of three literature searches: (i) a cited reference search of four key conceptual life course publications,^1^^,^^4^^,^^9^^,^^15^ and Medline searches for publications including the terms (ii) ‘life course’ and (iii) ‘developmental origin’ or ‘fetal origin’ (see Box 1).

**Box 1.** Reviewing the published literature

To assess developments in life course epidemiology since the inception of this term in 1997, we aimed to capture relevant papers published in this area up to the end of 2014 using two searches (undertaken in February and March 2015):

1. A cited reference search of four publications on life course epidemiology (from the date of their publication to 31 December 2014):
  -      I. Kuh D, Ben Shlomo Y (eds). *A Life Course Approach to Chronic Disease Epidemiology*. 1st edn. Oxford, UK: Oxford University Press,. 1997.
  -     II. Kuh D, Ben Shlomo Y (eds). *A Life Course Approach to Chronic Disease Epidemiology*. 2nd edn. Oxford, UK: Oxford University Press, 2004.
  -    III. Ben-Shlomo Y, Kuh D. A life course approach to chronic disease epidemiology: conceptual models, empirical challenges and interdisciplinary perspectives. *Int J Epidemiol* 2002;**31**:285–93
  -    IV. Kuh D, Ben-Shlomo Y, Lynch J, Hallqvist J, Power C. Life course epidemiology. *J Epidemiol Community Health* 2003;**57:**778–83
2. A free-text search to identify all literature indexed in Medline which:
  - included the term: life course (i.e. ‘life ADJ course’ or ‘life-course’ or ‘lifecourse’), in the title or abstract;
  - was published between 1 January 1990 and 31 December 2014;
  - was published in the English language and indexed as study of humans.

To enable us to draw comparisons with developments in the Developmental Origins of Health and Disease (DOHaD) we undertook a third search (in March 2015):

3. A free-text search to identify all literature indexed in Medline which:
  - included the term: ‘developmental origin*’ OR ‘fetal origin*’ OR ‘foetal origin*’ in the title or abstract;
  - was published between 1 January 1990 and 31 December 2014;
  - was published in the English language and indexed as study of humans.

The results of each of these searches were downloaded to reference management software and duplicates were removed. See Supplementary Data (available as Supplementary Data at *IJE* online) for number of search results returned.

Each result from search 2 was screened and classified by Y.B.S., R.C. or D.K. using standard criteria based on its relevance, type of paper, outcome, exposure and analyses, timing of exposures and setting.

We have, from the outset, highlighted the long-term history of life course concepts and the fact that disciplines outside epidemiology, such as demography, sociology, anthropology and psychology, have considered these broad ideas for longer.^3^^,^^9^^,^^16^^,^^17^ (see commentary by Alwin^18^). It was thus no surprise that our literature searches, even when restricted to Medline, returned empirical as well as narrative and review papers from journals in these disciplines as well as epidemiology and public health, highlighting the broad appeal of a life course perspective. Alwin^18^^,^^19^^,^^20^ discusses the conceptual and methodological developments and the growth of empirical evidence in these other fields and draws parallels with the developments in epidemiology.

**Figure 1.**
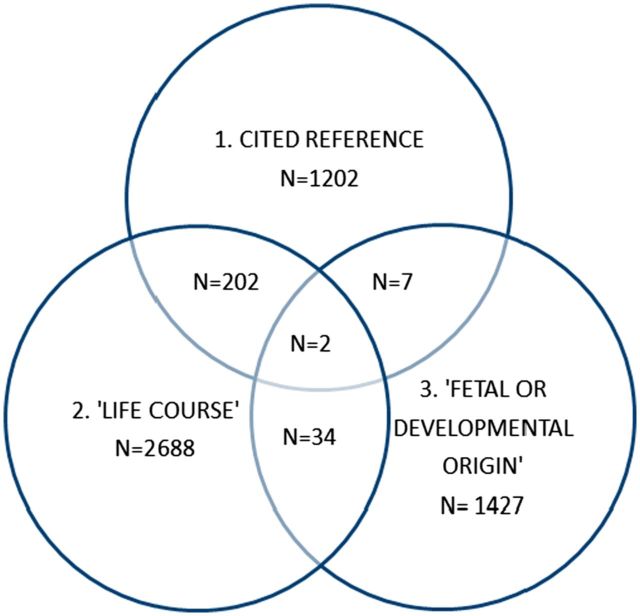
Venn diagram showing overlap between search results.

Life course perspectives in epidemiology were eclipsed in the mid 20th century by the almost exclusive focus on the adult lifestyle model of disease causation.^21^ The catalyst for the revival of life course thinking in epidemiology was the seminal work of David Barker and colleagues on the fetal origins of adult disease (FOAD)^22^ from the late 1980s, which was subsequently extended to the developmental origins of health and disease (DOHaD) at the turn of the century.^23^^,^^24^ The first of three phases in the development of life course epidemiology reflects this: a focus on the associations of early life factors with clinical disease endpoints. Life course epidemiology is often incorrectly interpreted as being synonymous with DOHaD, but there are both similarities and differences (see below) and interestingly there was only limited overlap found between publications in these two fields (see Figure 1).

We consider the second main phase in the development of life course epidemiology to be the expansion of the field to consider a wider range, and more integrated study, of outcomes, including different aspects of physiological function and their natural history across life, and of biological, psychological and social risk factors acting independently, cumulatively or synergistically across different life stages. These developments were possible because of the growing number of birth cohort and other longitudinal studies with increasingly rich data archives, and the development of life course methods that facilitated such analyses. The study of functional measures is discussed further below and this shift, alongside maturation of cohort studies with relevant data, has led to the third and most recent phase in the development of life course epidemiology; the application of a life course epidemiological approach to the study of ageing.

**Figure 2.**
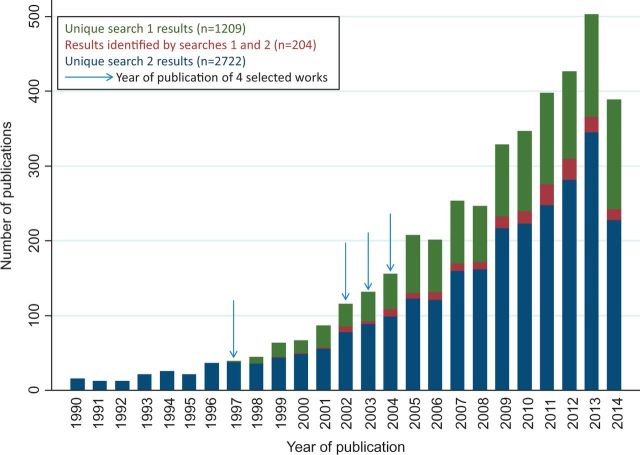
Number of publications identified by literature searches 1 and 2 by publication year.

There remains a healthy appetite for life course epidemiology after almost 20 years, and many of the ideas proposed and subsequently developed remain scientifically innovative and policy relevant. This is supported by evidence of a year-on-year increase in the number of papers that cite one of the four key references or use ‘life course’ in their title or abstract (see Figure 2). However, some caution is required as there has been a secular increase in the number of scientific publications in general and when, classifying abstracts, we observed that the term ‘life course’ is sometimes used loosely and occasionally inappropriately. This supports the assessment made by Alwin in his review of life course concepts in social sciences, that life course ‘has become a catch all term, taken to refer to all manner of different aspects of individual experiences in biographical time’.^18^^,^^19^ In addition, it was noted that a number of papers use the term ‘life course’ in their abstract despite not applying this approach; rather, they have concluded that this may be useful in future research. In contrast, some of our own papers, that we would describe as examples of life course epidemiology, were not captured by the searches, as titles and abstracts of epidemiological papers generally focus on reporting study design, exposures and outcomes and do not necessarily report the conceptual approach taken: a limitation with current structured abstracts and indexing.

Despite these caveats, we identified trends in the published literature which supported our observations on the key phases of development in the field, and also provided evidence of the balance between papers reporting empirical studies and other types of papers. In the case of empirical studies, we tried to capture the types of outcomes and exposures and the settings in which the studies took place. Key points are briefly summarized here, and relevant points and publications uncovered by these searches are cited throughout the paper. Approximately 60% of the classifiable papers identified by our Medline search for publications that included the term ‘life course’ in their title and/or abstract (i.e. search 2) reported quantitative empirical analyses (for breakdown of types of paper see Supplementary Data, available as Supplementary Data at *IJE* online).

Among these empirical papers there was evidence that studies of metabolic conditions (including obesity and diabetes), cardiovascular and socioeconomic outcomes were the most commonly investigated across all years; as were studies of mental health^25–27^ where there has been a longstanding parallel interest in the life course or life span perspective. There was evidence of a diversification in the types of outcomes being studied over time, with more recent empirical papers reporting life course analyses of a wider range of outcomes including cancer, musculoskeletal disorders, disability, oral health, injury, infectious diseases, migration, genetic and epigenetic markers and age-related phenotypic trajectories. There were also a number of papers reporting on behaviours as outcomes: here cigarette smoking, alcohol consumption and physical activity were common, but other topics included marijuana use and criminal behaviours, with several investigating these behavioural trajectories over different life stages.^28–31^

In terms of exposures, there has been an increase over the past 10 years in the proportion of empirical studies which model exposures as repeat measures or trajectories covering multiple life stages. The impact of an adverse socioeconomic environment in childhood and adulthood on later outcomes remained an enduring theme, perhaps because such exposures more easily lend themselves to being modelled within a life course framework, have been collected relatively frequently across life and can be retrospectively ascertained by a variety of methods. The impact of early psychosocial environment, gender, body size and shape and behavioural risk factors also appear to be persistent topics. In terms of setting, the majority of studies are based in the UK, mainland Europe and the USA, which has been facilitated by the availability of data from maturing birth and historical cohort studies, particularly in the UK.^32^ More recently, there has been an increase in the number of studies set in other high-income countries, and also a rise in relevant studies being conducted in low- and middle-income countries (see commentary by Tollman and colleagues^33^). The broadening of study settings is essential to help establish which life course associations are contextual and which are universal. A small but increasing number of papers involved multiple cohorts (see below) which sometimes crossed several of these settings.

## From FOAD to DOHaD and the life course

We have always acknowledged the importance of the ‘Barker hypothesis’ or FOAD model as a stimulus for life course epidemiology. We viewed this as one set of competing and innovative aetiological models that needed to be tested alongside others. In addition, as population scientists, understanding temporal, geographical and social differences in disease risk is important, as well as elucidating more fundamental downstream mechanisms that may explain epidemiological associations. We note that the term ‘life course’ has gained popularity in the DOHaD world (anecdotal observation at the Eighth World Congress on DOHaD, Singapore, 2013) and the term ‘life course biology’ has been used by DOHaD colleagues,^34^ so it is important to understand what are the substantive similarities and differences in approaches.

The DOHaD model proposes that responses to the impact of any environmental challenge can be seen to operate over different time scales: (i) homeostasis is the immediate response; (ii) predictive adaptive response (PAR) occurs in earlier life and maximizes the chances of survival and reproduction; and (iii) natural selection occurs over many generations.^24^^,^^35^^,^^36^ Chronic disease may arise where a PAR is maladaptive in the long term, e.g. a PAR to a nutrient-poor environment in childhood will be counter-productive if the adult environment is obesogenic and so could lead to type II diabetes. This is analogous to the ‘antagonistic pleiotropy’ model of ageing, whereby pleiotropic genes with good early effects would be favoured by selection even if these genes had bad effects at later ages as long as the organism had successfully passed on these genes to future generations.^37^ The ‘live fast, die young’ strategy is successful when there are strong external influences on mortality so that few survive much beyond reproductive age. Both models assume that survival into later life has no benefits on the survival advantage of first- or even-second generation offspring. This may not be true for human societies where kinship networks can play an important role in the health and well-being of children and grandchildren, especially in low- and middle-income countries where the ravages of HIV infection have left generations of children dependent on grandparents for care. We agree with a recent DOHaD review paper^37^ which argued that the early use of the term ‘programming’ was unhelpful in implying an overly deterministic view of DOHaD, and was not supported by the empirical evidence.

Scientists concerned with life course epidemiology or the DOHaD approach share many common research interests, especially in understanding how developmental factors are associated with adult function and disease, but there are important differences. Life course epidemiology is equally interested in the maintenance of function or its rate of decline post maturity and the factors across the whole of life that determine this. The focus of DOHaD is on developmental mechanisms within an evolutionary framework, but whereas this framework is a scientific strength and is widely relevant to many outcomes, research has focused on the developmental origins of chronic diseases, especially cardiometabolic diseases. In contrast, life course epidemiology with its public health background remains interested in both social and biological pathways, and has been applied to a wider variety of outcomes, as evidenced by our literature searches. DOHaD, despite its growing interest beyond the immediate postnatal period, has focused on interventions in young and/or pregnant women or in infancy, generally with a biomedical emphasis, and has been somewhat dismissive of the potential for later life interventions to modify disease risk or alter disease progression. A life course approach is essentially agnostic and tries to provide empirical evidence to guide the most appropriate timing of any intervention, be it at a biomedical, individual or societal level, to maximize population health. The DoHAD approach has been more successful in engaging the basic science community, but the life course approach has also benefited from interdisciplinary interactions with basic scientists: for example, research relating to the insulin-like growth factor cancer inter-relationship and how it has influenced life course studies in human populations (summarized in reference^12^). The commentary by Boyce and Shonkoff^38^ reinforces the need to triangulate life course epidemiology with mechanistic insights from biology. We believe that both approaches have much to offer each other, despite the limited overlap in the literature to date (see Figure 1), and anticipate further convergence in how ideas from both camps can be used to cross-fertilzse future research (see commentary by Hanson and Gluckman for further discussion^39^).

## Revisiting life course models

In our original work we proposed several conceptual models such as critical/sensitive period models, as exemplified by DOHaD, and accumulation or chains of risk models which are more common in the social epidemiology literature. We illustrated these ideas graphically using simplified causal diagrams, though at this time we were unaware of the growing literature on directed acyclic graphs (DAGs),^40^^,^^41^ (see commentary by De Stavola and Daniel^42^ for a more detailed discussion of causal inference in relation to life course concepts and VanderWeele’s unified approach to decomposing exposure outcome relationships in the presence of mediators and potential interactions^43^). Initially these critical period and accumulation models were presented as different classes of models, though we have since noted^11^ that it is more sensible to view critical or sensitive period models as special sub-sets of an accumulation model when considering the effects of an exposure over time—hence, the effects of exposures over different time periods do not add up in a simple additive fashion but differ in the strength of effect in a quantitative sense (sensitive period) or in a qualitative sense, so that they can only be observed in one time period (critical period) (a point also made by Alwin^20^).

**Figure 3.**
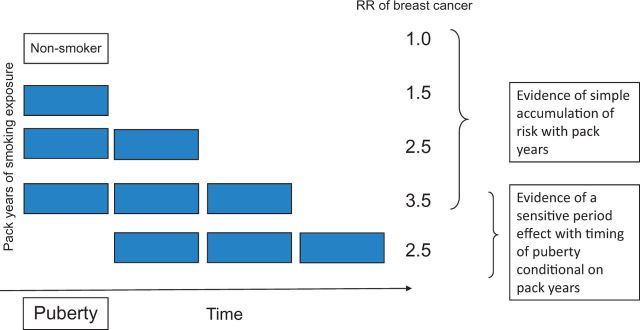
Testing for evidence of accumulation or sensitive period effects in models of the association between smoking pack-years and risk of breast cancer.

In our initial work, we had conceptualized an accumulation of risk as the incremental burden of different exposures with age, and highlighted the issue of clustering of exposures. However, our literature searches highlight that most papers that try to address this model actually use the cumulative exposure of a single exposure, such as periods of adverse social circumstances, in a quantitative fashion. This is a standard epidemiological approach (‘dose-response effect’) and of particular interest to occupational or environmental epidemiology. Even here, arguments persist as to how best to model the functions of intensity and duration.^44^ A life course approach highlights the importance of timing over and above cumulative exposure. We have illustrated this with a simple figure (see Figure 3) examining the relationship between pack-years of smoking exposure and breast cancer risk. A conventional dose-response model would be consistent with an accumulation model. However, it would fail to recognize a sensitive period model if it did not consider whether initiation of smoking during puberty or post-pubertally altered the risk conditional on smoking intensity and duration.^45^ Of course there may be other potential mechanistic pathways so that early and intense smoking may itself predict exposure to smoking at the time of disease onset, and hence the induction may be relatively short but predicted by early smoking behaviour.

Many contemporary papers have formally modelled trajectories of exposure, especially those examining anthropometric exposures, using a variety of statistical approaches (for example two-stage spline models, latent class models, multilevel models, SITAR—see reference Tu *et al.* 2013^46^ for a review of this area and accompanying commentary by Hardy and Tilling^47^) to see whether different exposure patterns are associated with risk. As an exemplar, one can then pose the question as to whether the chronic disease risk associated with midlife obesity is different for those sub-groups who have always been at the upper end of the body mass index (BMI) distribution and have tracked into adulthood, versus those who were born underweight and gained weight in childhood or early or mid adulthood. An alternative has been to use a Mendelian randomization approach which uses genetic markers as instruments for behavioural or phenotypic variability, thereby exploiting the random allocation of parental genotype and avoiding issues of confounding and reverse causation.^48–50^

The notion of a critical/sensitive period was initially framed around early life, stimulated by FOAD. It is increasingly being shown that biological and social transitions during other life stages are also likely to be sensitive periods where adverse exposures may have more impact than at other times, as birth cohort studies have matured and cohorts with rich data on adolescence, young adulthood and mid life^51–53^ have emerged. For example, the timing of the pubertal growth spurt appears to be important for levels of insulin-like growth factor-I,^51^ breast cancer risk^54^ and trabecular volumetric bone mineral density.^55^ Accelerated increases in blood pressure across early and mid adulthood are associated with a greater left ventricular mass index and indicators of diastolic dysfunction in early old age.^56^ Identifying sensitive periods can be difficult, but will be helped by a better understanding of phenotypic/functional trajectories (see below), as inflection or turning points in a trajectory may highlight the greater value of an intervention, assuming this is at least partially modifiable and driven by concurrent exposures.

Life course models have also stimulated statistical methods to help differentiate between various competing models. These have used nested models,^57^ causal inference algorithms,^58^ least-angle regression,^59^ structural equation models^60^ and marginal structural models.^61^ Any life course model which considers exposures, confounders and potential intermediaries is bedevilled by the problem of time-varying confounders and ‘intermediary confounders’,^62^ i.e. a confounder of a mediator which is itself affected by the exposure and hence also acts as an intermediary. This problem is not specific to life course epidemiology but is obviously more problematic when examining exposures acting over a long time period, for example when testing whether cognitive function in adulthood is associated with mortality rates, given the large number of social and behavioural covariates that could act in several different ways.^63^

## The drivers and consequences of functional change

The original life course models generally assumed a fixed outcome, such as disease diagnosis at a point in time. However, as noted above, lifetime trajectories of functional phenotypes are increasingly being studied as outcomes. This shift in focus has been driven in part by our initial work which put forward hypothetical phenotypic trajectories, adapted from Strachan’s review of lung function in the original life course textbook.^64^ These illustrate how the same disease endpoint could be reached through either suboptimal development or accelerated decline or a combination of both. At that time these patterns were hypothetical, but recent data have identified that around half of all cases of chronic obstructive pulmonary disease occur in individuals with suboptimal adult lung function but normal age-related decline, whereas the other half occur after normal development of lung function with an accelerated decline phase.^65^ This simple figure highlighted two important messages: (i) that epidemiologists should consider continuous traits, such as lung, cognitive or muscle function and complex phenotypes such as ageing, as well as binary phenotypes; and (ii) that classifying disease endpoints at one time period, usually in later life, ignores the underlying trajectories, thereby increasing phenotypic heterogeneity. This will attenuate risk factor associations if they are only of causal relevance for some sub-groups but not others. The same argument has been made for understanding phenotypic variability in relation to genetic association studies.^66^ Using functional outcomes also has the benefit of facilitating the study of preclinical disease and identifying high risk sub-groups through risk stratification; functional outcomes may have more statistical power; they demonstrate the dynamic nature of disease aetiology with periods of recovery as well as loss; and they can be used for clinical triage. Being able to detect early markers of suboptimal development and/or an accelerated trajectory of functional decline before it is clinically manifest is fundamental to a life course perspective, because it offers an opportunity to delay the rate of decline and/or the onset of functional impairments through preventive strategies. How these trajectories differ with person (age, gender, social circumstances), place (geography) and time (birth cohorts) may all provide important mechanistic and aetiological clues for further investigation.

Where the drivers of functional change are themselves changing over time, joint modelling of both trajectories of exposure and phenotype and their inter-relationship is required.^67^ For example, data from the ALSPAC birth cohort has been used to model how maternal change in blood pressure during pregnancy may relate to offspring blood pressure trajectories between 7 to 18 years, using bivariate linear spline models.^68^ In this case there is no problem with temporality, as offspring trajectories cannot influence maternal patterns. However, when studying functional trajectories that may be occurring simultaneously, for example cognitive and physical function, it is unclear which should be regarded as the driver and which the consequence; indeed both may drive each other.^69^ The hypotheses to test may be taken for theoretical and pragmatic reasons, such as the ages when data points are available; however, interpretation of findings needs to acknowledge the inherent challenges of identifying the temporal ordering in these situations.

The research evidence on functional change within a life course context is less developed (see this reference^70^ for evidence of change in cardiometabolic outcomes). It has been harder and more costly to achieve, due to the requirements for multiple and frequent measures, though this may be changing due to technological innovations. Not surprisingly this has resulted in gaps around several important questions. So, for example, to what degree do earlier life factors that have been previously shown to be associated with the level of adult function, also drive functional change, or is the association merely due to the maximal level attained through developmental factors? Smaller birthweight is associated with higher blood pressure but this does not appear to amplify with age, suggesting that the age-related increase is similar regardless of birthweight.^70^^,^^71^ In contrast, early adverse social circumstances are associated with greater increases in blood pressure in mid life,^72^ perhaps through an influence on BMI gain.

One of the inherent problems of characterizing lifetime functional trajectories and their inter-relationships is the need for longitudinal data across the whole life course, which are rarely available. Alternatives include the use of cross-sectional data from a single study with a very wide age range, or the creation of a synthetic birth cohort by combining cross-sectional and/or longitudinal data from multiple cohorts covering different age ranges usually with some overlap. Both approaches have been undertaken to establish normative data on grip strength across life.^73–75^ To understand within-person change, one must combine longitudinal data across different ages as was undertaken by Wills *et al.*^76^ using blood pressure data from eight British cohort studies. Interestingly, participants in the only occupational cohort (Whitehall II study) had a more moderate/less marked mid life increase in blood pressure, suggesting modifiable social determinants, unless this was due to selection bias. By drawing together data from different cohorts, alcohol consumption patterns have also been shown to change with age in a similar way for men and women, but men have a higher peak level.^77^

By concatenating data from overlapping age groups from different cohorts,^73^ or simply using cross-sectional data covering a wide age range,^74^^,^^75^ we are assuming that there are no period or cohort effects which may not be true. This can be formally tested when different birth cohorts with data across the same age periods are directly compared with each other. Johnson *et al.*^78^ harmonized repeat measures of body size from five British birth cohorts from the CLOSER consortium, born in 1946, 1958, 1970, 1990–92 and 2001, and showed that the probability of being overweight or obese increased rapidly after childhood in the oldest three cohorts; the probabilities of overweight in the two younger cohorts at 11 years were already double those in the three older cohorts; and the shifts in age-related overweight or obesity trajectories across these cohorts was due to BMI being positively skewed in the more recent cohorts. The earlier onset of obesity^78^ and hence greater obese life-years^79–81^ in more recent cohorts can be used to predict future disease burden and demands on health service delivery. Such cross-cohort collaborations with re-analysis of individual participant data are extremely valuable, but technical issues around data harmonization as well as the important assumptions when aggregating data should not be under-estimated.

## The challenge of ageing research for life course epidemiology

Applying life course ideas to ageing research has made us reconsider and modify some of our earlier, simpler life course models. How one defines and measures ageing remains debated, with much interest in developing biomarkers^82^ of biological as opposed to chronological age. These can be single biological markers of a cellular process, e.g. telomere length or epigenetic clock,^83^ but are more often composite markers that aggregate determinants and/or consequences of ageing.^84^,^85^ In each case the aim is to demonstrate that the marker has added value in predicting other outcomes, such as mortality, morbidity or function, over and above chronological age. The predictive value of these markers may differ at different stages of the life course and may not necessarily remain constant over secular period; and as with any other markers of continuous traits that show population variation and age-related changes, they should be studied from a life course perspective.

We have noted that there is a lot of confusion about what is meant by the term ‘healthy ageing’, partly because researchers are trying to do two things. Some researchers include any dimension of the health and well-being of older people regardless of whether the health dimension under study changes adversely with age. Others, including ourselves,^5^ separate out healthy biological ageing from how people feel or function socially, preferring to study their bi-directional relationships rather than derive some composite healthy ageing index. Ageing is expressed as a failure to maintain structural or functional integrity, leading to system failure and ultimately death. Healthy biological ageing covers three key longitudinal components: survival to old age, delay in the onset of age-dependent diseases, and maintenance of optimal functioning for the maximum period of time. We distinguish three levels of functioning that all generally show a decline with age: capability describes physical and cognitive functioning at the individual level, and is defined as the capacity to undertake the physical and mental tasks of everyday living; the physiological functioning of each body system; and the underlying functioning of molecules and cells. Studying the lifetime trajectories of these different aspects of functioning, their dynamic interplay over time, and their lifetime environmental and genetic determinants has become a major focus in life course epidemiology.

**Figure 4.**
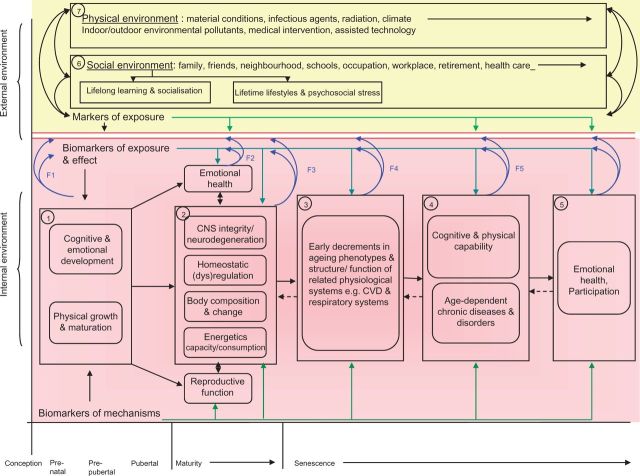
An integrated life course model of ageing. Taken from: Kuh D, Cooper R, Hardy R, Richards M, Ben-Shlomo Y. *A Life Course Approach To Healthy Ageing*. Oxford, UK; Oxford University Press, 2014.

Ferrucci and Studenski^86^ have proposed a model for understanding frailty or healthy life expectancy that focuses on four key ageing phenotypes: (i) central nervous system (CNS) integrity or neurodegeneration; (ii) homeostatic regulation or age-related dysregulation in signalling pathways that maintain homeostasis (encompassing both the endocrine and immunological systems); and age-related changes in (iii) body composition (muscle, fat and bone) and (iv) energy capacity and consumption. We have incorporated these themes into an integrated life course model of ageing (see Figure 4). We have also added reproductive ageing (not normally considered by gerontologists), given the importance of development and reproduction from an evolutionary perspective, and emotional health which itself may be a determinant and/or consequence of ageing but is important for social participation and quality of life. A woman’s physiological response to the challenge of pregnancy and childbirth provides insights into adaptive capacity, and reproductive characteristics (such as gestational diabetes, offspring low birthweight and early menopause) that may act as sentinels for later disease and accelerated ageing.^87^

We delineate six phases of life (conception, pre-natal, pre-pubertal, pubertal, maturity and senescence) though we recognize the importance of inter-generational influences through genetic, epigenetic or social transmission. The other dimensions are what we refer to as the external and internal environments, the former referring to the social and physical milieu which will in turn influence and interact with the latter. This may operate via: direct physiological change, e.g. weight loss due to famine; or through internal adjustment or modification, e.g. down-regulation of the growth hormone insulin-like growth factor axis in relation to dietary exposure,^88^ analogous to the PAR with potentially long-term adverse or beneficial consequences.

Not surprisingly these are dynamic systems with feedback loops that can operate in both negative and positive ways to either maintain stability and homeostasis or to increase deviation and instability, a feature seen in ageing systems. Our own work^89^ used simple epidemiological measures of physical and cognitive capability and of emotional wellbeing, as these are easy to measure in large-scale epidemiological studies. Comorbid disease such as chronic obstructive pulmonary or cardiovascular disease will usually impact on these measures as well as influencing body composition and energetics. CNS integrity is clearly important for cognitive function but also plays roles in physical capability through neuromuscular coordination and through endocrine and autonomic pathways. The ‘common cause hypothesis’ argues that ageing is associated with decline across multiple systems through common aetiological mechanisms, be they genetic or environmental. This may not always be evident, as the rate of decline may differ across domains so that one system, e.g. cognitive decline, may appear to be more dominant even though physical decline is also present. Some evidence exists for the common cause hypothesis, though a recent systematic review^69^ found only modest evidence and also issues around comparability between studies.

The stochastic nature of ageing means that this integrated life course model is unlikely to predict individual outcomes with much certainty, but it may explain group differences in ageing.^90^ This generic model had three phases: (i) growth; (ii) plateau; and (iii) degeneration, analogous to Baltes’ allocation of resources across the life course—with allocation of resources towards growth in childhood, maintenance and recovery (resilience) in adulthood and regulation or management of loss in old age^91^ (see also commentary by Alwin^18^). This simple pattern may not always apply; for example, systolic blood pressure shows an accelerated increase with age with a possible decline by the eighth decade.^76^ It is possible to reconcile this divergent pattern by reconceptualizing the phenotype to be arterial elasticity, rather than systolic blood pressure. This will decrease with age, hence resulting in increased systolic blood pressure. The apparent decrease in blood pressure in later life, assuming this is not a treatment effect, is not due to increased elasticity but more likely reflects a different pathophysiological mechanism such as declining autonomic control on vascular tone. However, this generic pattern may not be true for all systems and structures. Cross-sectional and longitudinal MRI scans of the brain suggest more variable patterns. Brain volume appears to increase in the first part of life, presumably due to myelination and axonal growth but, before the maturational changes are complete, degeneration due to selective loss of small-diameter myelinated axons (primary degenerative event) and demyelination of larger connections (secondary degenerative event) occurs. The superimposed effect of both processes result in an early period whereby the volume increase slowly decelerates and a later period, where volume reduction begins to level off.^92^ Different brain structures, however, showed different trajectories; the hippocampus showed a plateau and then accelerated loss pattern whereas the cerebral cortex had a simple linear decline (at least from age 20 years).

## Physiological, psychological and social resilience

We know there is great heterogeneity in biological ageing and our models, to be of any value, need to be able to take into account this variation in resilience to environmental challenges with age. The ability to repair and compensate was referred to by Martin^93^ as ‘sageing’, though he considered this to only occur in the phase between that of the mature, reproducing adult and the senescing adult. We would argue that this view is too limited and in fact this can occur throughout life though the relative ease and ability to compensate may well differ (sensitive periods) and is generally regarded to decline with age (known as ‘homeostenosis’).^94^ A life course approach is particularly interested in understanding the relationship between the growth, plateau and degeneration phases. Are they independent or do similar or different risk factors determine them? Is there an interaction between these phases and clinical disease or functional limitations?

**Figure 5.**
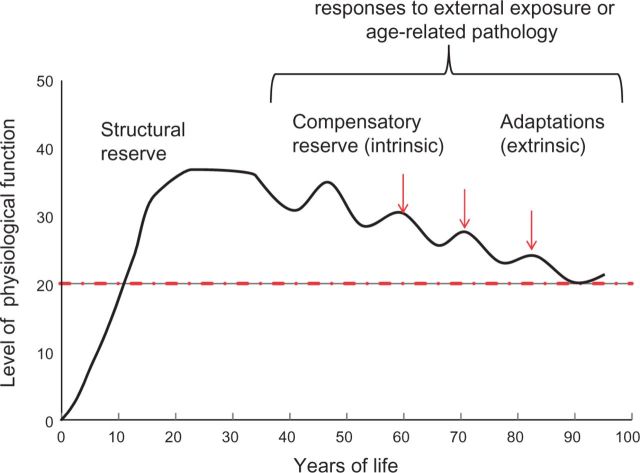
The influence of structural and compensatory reserve and adaptations on a life course trajectory.

One obvious relationship is the role of what we have previously described as structural reserve; those with better development of a phenotype (e.g. lung function, bone mass, cognitive capability) will have further to decline before reaching any impairment threshold and hence will present with disease at a later age, or may never present if they die of another cause. In our original figure this was illustrated by the peak level of function at the plateau phase. We failed to illustrate the ability to recover from adverse exposures and how this changes with age, what we now term as compensatory reserve (see Figure 5). Although such short-term responses will be beneficial, there may or may not be longer-term costs in relation to ageing. McEwen and Wingfield described this longer-term adverse effect as type 2 chronic allostatic load.^95^

Thus, the degree of heart failure subsequent to a heart attack will not only depend on the size of the infarction and how healthy it was before this event (premorbid state) but also the post-ischaemic response in generating collateral circulation thereby salvaging cardiac muscle and function. Intrinsic compensatory reserve may or may not be related to structural reserve, an empirical question that requires attention. For example, does recovery post stroke relate to developmental pre-stroke synaptic connectivity or does it merely reflect acute central repair processes? Similarly, is age-related loss of immune responsiveness to novel antigens related to the degree of immunostimulation in early life? In order to capture adaptive responses, it is necessary to physiologically stress the system. This may be a natural process, as seen in the rise in cortisol levels on waking (cortisol awakening response) or through a standardized exposure such as the oral glucose tolerance test to measure glucose and insulin responsiveness to a standard intake. Few studies have made use of other opportunistic dynamic exposures, such as immune responsiveness to flu vaccination,^96^ blood pressure decline in response to antihypertensive agents, or recovery from an acute event such as illness or surgery. A recent interesting exemplar was a study of the impact of acute stroke on longitudinal cognitive trajectories.^97^ Three distinct patterns emerged: (i) acute decline but no change in age-related decline (seen with word learning list and recall); (ii) accelerated age-related decline (seen with executive function); and (iiii) both acute decline and accelerated age-related decline (seen with global cognition).

Old age is obviously associated with greater morbidity often necessitating major health care interventions. Recent evidence highlights the predictive value of the pre-admission physical trajectory in predicting 1-year survival after an acute illness resulting in an intensive care unit episode.^98^ Greater understanding of how functional trajectories predict clinical outcomes would enhance precision medicine. Better selection of those who have most to benefit from an intervention would not only reduce health care expenditure but also avoid unnecessary and often invasive procedures for patients and families. Other predictors of post-surgical outcomes also illustrate the often paradoxical nature of mid life risk factors in older populations. Being overweight and obese in mid life increases premature mortality risk, but at older ages both are associated with reduced postoperative mortality compared with normal weight after elective joint replacement.^99^^,^^100^ Whether this reflects a healthy survivor effect, confounding by comorbidity^101^ and smoking status or a genuinely protective effect of greater lean mass which is positively correlated with fat mass, in response to the acute trauma of surgery, remains to be determined.

In parallel, how an individual responds to adversity will result in extrinsic compensatory adaptations which may be facilitated or hindered by the external environment. Baltes proposed a meta-theory known as the selective optimization and compensation (SOC) model to understand human development. In addition, he stated ‘with age, and conditioned primarily by the negative biological trajectory of the life course, the relative power (effectiveness) of psychological, social, material and cultural interventions wanes.’^91^ This ability of individuals to optimize and compensate will be determined by a variety of factors; psychological trait (self-efficacy), educational resources (health literacy), material resources (socioeconomic position), familial and social resources (inter-connectedness) and geographical factors acting on the household, neighbourhood and locality. For example, higher socioeconomic position, social networks and residential location may facilitate engagement with intellectual pursuits which in turn could maintain synaptic connectivity through cognitive stimulation, and hence slow down decline or maintain cognitive function independent of cognitive reserve, in the presence of underlying neurodegeneration.

## Variability over varying timescales

The other limitation of our illustration of different trajectory patterns is that this merely represents the mean trajectory and fails to illustrate variability (measured over seconds, minutes, hours, days, weeks) which may provide added predictive value for phenotypic integrity. Blood pressure variability for a given identical mean level independently increases risk of cardiovascular disease,^102^ possibly because it is a marker of arterial stiffness which predicts cardiovascular morbidity independent of mean blood pressure^103^ or is also a marker of failing central homeostatic mechanisms that maintain blood pressure within a limited range. Similar observations have been seen for gait speed in older populations in which a larger standard deviation of gait speed rather than mean level predicts future falls risk^104^ and probably reflects underlying neurological dysfunction. Past epidemiological studies have not been able to capture variability, especially not on a day-to-day basis, because of the prohibitive cost and respondent burden except in small-scale detailed studies. New technologies now make this a feasible proposition although our understanding of the life course determinants of such compensatory responses is currently limited. Whether these are modifiable is clearly important; we know from the LIFE trial that increased physical activity in high-risk older people can reduce the incidence of mobility disability, although this was not associated with a reduction in hospitalization.^105^

## Current challenges of life course epidemiology

This review has shown that the field of life course epidemiology has a healthy scientific agenda focused on understanding how different aspects of function change (at the individual, system and cellular levels) across life, and on discovering their lifetime risk factors, mediators, modifiers and consequences. Much of this agenda is shared with our colleagues in DOHaD, in ageing research (see commentary by Newman^106^) and in geroscience (see commentary by Ferrucci^107^^,^^108^) and we believe our complementary approaches add value and hence should be further integrated. This scientific agenda attracts researchers from many disciplines, and the demographic pressures of an ageing population on health and social care should ensure a parallel political agenda on the importance for these research streams.

A healthy scientific agenda ensures continuing technological developments which in turn provide new opportunities for life course epidemiology and for epidemiology more generally. We give a few examples. Technology or ‘digital epidemiology’^109^ increasingly allows us to capture real-time dynamic assessments of function, environment and behaviour which provide additional information on short-term variability within the context of long-term change. There are growing opportunities for exploiting record linkage, assuming ethical issues and consents have been addressed, which reduce respondent burden and inherent problems of self-reporting. This has additional added value in enhancing analyses that incorporate missing data, always an in issue in any longitudinal study, and help produce less biased estimates of effect as seen recently with data from the ALSPAC study.^110^

A priority is to find ways to capture the care pathways and the interactions between individuals and their experience of health and social care that may shape future functional trajectories; for example, how dynamic events, such as acute illness and the care received at that time, punctuate trajectories of decline in later life.^111^ Life course and longitudinal methods are being used to harness the ‘omics’ revolution to this scientific agenda in the hope that new aetiological insights into underlying biological pathways linking earlier exposures to later function will be generated^112^ and new quantitative biomarker profiles will predict age-related functional change and disease development (also see *IJE* special editions on epigenetics and Mendelian randomization^50^^,^^113^). Any future review of life course epidemiology will need to evaluate the success of these approaches in addressing these scientific questions.

Past, current and future developments in life course epidemiology and in life course cohorts are intimately intertwined: since the 1990s, there has been a ‘feast’ in terms of the growing number of cohorts with increasingly rich data archives (social and biomedical) for testing life course hypotheses; this has helped to maintain and continually evolve the scientific agenda (see editorial by Brayne^114^). We see a number of challenges ahead for cohort studies in a time of financial austerity. Each cohort study must justify its scientific niche, as well as maximize its value from cross-cohort collaborations, especially with the rise of large biobank studies. In the ideal world, both types of study are required as they are complementary, but in the real world they compete for limited resources. Recent cuts to cohort funding and/or challenges in participant recruitment are cause for concern^115^^,^^116^ and will do little to attract a new generation of primary investigators to invest in cohort management, collect and assess data quality, and incorporate new life course methods of statistical analysis so that the benefits of longitudinal data on more granular exposure and outcome data can be best realized.

Another challenge is to translate the growing body of evidence on the lifetime influences on adult health, disease and ageing into practice or policy-relevant guidelines and intervention studies to improve the population health^117^ (see commentaries by Boyce and Shonkoff^38^ and Aihie Sayer and Gill^118^). Strategic and policy documents increasingly espouse a life course perspective and cite evidence from cohort studies,^119^ though this is usually within an early life remit focusing on maternal and child health,^117^^,^^120^ both important areas in their own right. However, some research funders, policy makers and governments consider that the translational agenda is not moving fast enough. Life course epidemiology has mainly relied on observational studies or, rarely, the opportunistic long-term follow-up of randomized controlled trials.^88^^,^^121^^,^^122^ It may also be that the implications from life course research, for example about the long-term effects of adverse socioeconomic and psychosocial environments, may not be attractive to politicians who seek short-term wins that are achievable within an electoral cycle. Finally it is important to remember that some life course findings may be highly context specific, in both time and place, making generalizing from observations to policy formulation problematic. Systematic reviews and cross-cohort comparisons are important and necessary pre-requisites before assuming immediate policy relevance.^123^^,^^124^

An eminent professor of epidemiology once commented, after one of our presentations, that whereas they really liked the concepts underpinning life course epidemiology, they were concerned that the data and analytical demands in testing these concepts may make our goals unachievable. We have always shared this concern but have been encouraged by the enthusiasm of researchers in rising to this challenge. We believe that in this new era of ‘big data’ and genuine multidisciplinary research groups with new disciplinary partners, such as computer scientists, engineers and mathematicians, we are in a far healthier place to take on these complexities. The new discoveries emerging from the ‘omics’ revolution need to go hand and hand with historical, current and future cohort studies, thereby allowing us to understand the interplay of molecular, system, individual and societal factors on development and maintenance of health and function as well as disease aetiology.

## Funding

R.C. and D.K. are supported by the UK Medical Research Council (programme codes: MC_UU_12019/1 and MC_UU_12019/4). YBS was supported by the NIHR School for Public Health Research (SPHR). The views expressed are those of the author(s) and not necessarily those of the NHS, the NIHR or the Department of Health. NIHR SPHR is a collaboration between: the Universities of Sheffield, Bristol, Cambridge, Exeter, UCL; The London School of Hygiene and Tropical Medicine; the LiLaC collaboration between the Universities of Liverpool and Lancaster and Fuse: The Centre for Translational Research in Public Health, a collaboration between Newcastle, Durham, Northumbria, Sunderland and Teesside Universities.

**Conflict of interest:** None declared.

## Supplementary Material

Supplementary Data

Supplementary Data

Supplementary Data
