# Supplementary material for: The last two decades of life course epidemiology, and its relevance for research on ageing
Source: Int J Epidemiol. 2016 Oct 6;45(4):973–88. doi: 10.1093/ije/dyw096 (PMC5841628; doi:10.1093/ije/dyw096)
Supplement: Supplementary Data [file dyw096_supplementary_data.zip › ije-2015-10-1317-File003.docx]

**Appendices**

**Supplementary Figure 1: Flow diagram of literature search results**

1. Results from cited reference search II

N=299

1. Results from cited reference search I

N=387

3. Results from free text DoHAD Medline search

N=1470

2. Results from free text life course Medline search

N=2926

1. Results from cited reference search IV

N=318

1. Results from cited reference search III

N=599

Duplicates removed

N=43

Total no. of unique references

N=5562

Duplicates removed

N=204

No. of unique references (searches 1 and 2)

N=4135

Duplicate search results removed, N=190

Total no. of unique citations

N=1413

**Supplementary table 1: Summary of findings from classification of abstracts identified by literature search 2 (i.e. Medline search for papers using the term ‘life course’ in their title and/or abstract (1990-2014), see text box for details of this search)**

|  | **%** |
| --- | --- |
| **Type of paper** (N=2196^a^)  Empirical  Methods – statistical or study design  Conceptual, narrative or qualitative reviews  Systematic review  Policy  Other  Unclassifiable | 59.7  5.9  27.4  1.6  3.5  1.5  0.5 |
| **Outcomes^b^** (N=1694^c^)  1. Infectious and parasitic diseases  2. Cancer  3. Blood and blood-forming organs  4. Endocrine, nutritional and metabolic  5. Mental and behavioural  6. Nervous system  7. Eye, ear & related structures  8. Cardiovascular system  9. Respiratory system  10. Digestive system  11. Skin and related structures  12. Musculoskeletal system  13. Genitourinary system  14. Pregnancy, childbirth & the reproductive system  15. Conditions originating in the perinatal period  16. Injury, poisoning and other consequences of external causes  17. Self-reported general health measures  18. Social outcomes  19. Multiple biomedical outcomes  20. Multiple biomedical and social outcomes  21. Mortality  22. Behavioural risk factors  23. Other | 0.5  3.3  0.8  9.7  21.9  1.5  0.5  9.7  1.2  2.5  0.1  4.6  0.8  5.1  0.4  1.7  7.5  8.7  4.0  1.2  5.4  9.9  11.6 |
| **Exposures** (N=1477^c^)  Socioeconomic environment  Psychosocial environment (extrinsic)  Physical environment  Cognitive  Body size and shape and maturation  Emotional (intrinsic factors)  Behavioural risk factors (incl. diet, activity, smoking)  Genetics  Epigenetics  Other | 41.5  22.1  2.7  3.9  12.6  7.4  11.3  3.6  1.8  28.4 |
| **Timing of exposures** (N=1239)  Pre-adult (<20 y)  Adult (≥ 20 y)  Both pre-adult and adult  Intergenerational | 16.0  29.5  50.2  4.3 |
| **Type of analyses** (N=1135)  Associational study covering 1 life stage^d^  Associational study covering ≥ 2 life stages^d^  Repeated measures/trajectories modelled | 23.9  60.2  16.0 |
| **Setting^e^** (N=1230)  UK  North America (incl USA and Canada)  Mainland Europe  Other high income country  Low to middle income country | 20.7  44.6  18.0  7.4  9.3 |

^a^ All 2926 abstracts identified in the original search were screened by YBS, DK or RC and those that were eligible underwent further classification using pre-defined criteria.

Papers were eligible for classification if the authors had self-defined their work as taking a life course approach (even if this would not meet the review authors’ criteria).

Papers were classified as not eligible (N=730) if the term life course had been used in the title or abstract in a different context and there was no evidence that the paper had any relevance to the life course approach as defined by Kuh and Ben-Shlomo.

Ns vary as it was not possible to provide a classification under each heading for all eligible papers.

^b^ The list of outcomes was defined based on WHO International Classification of Diseases (ICD-10) and International Classification of Functioning, Disability and Health (ICF) classifications, as follows:

1. Infectious and parasitic diseases

2. Cancer

3. Diseases of the blood and blood-forming organs and certain disorder involving the immune mechanism/functions of the haematological and immunological systems/structures of the immunological system

4. Endocrine, nutritional and metabolic diseases (incl. diabetes)/functions and structures of the digestive, metabolic and endocrine systems

5. Mental and behavioural disorders/mental functions

6. Diseases of the nervous system/structures of the nervous system

7. Diseases of the eye and adnexa/ Diseases of the ear/sensory function/pain/Eye, ear & related structures

8. Diseases of the circulatory system/functions & structures of the CV systems

9. Diseases of the respiratory system/functions & structures of the respiratory system

10. Diseases of the digestive system (includes dental health)

11. Diseases of the skin /functions and structure of the skin and related structures

12. Diseases of the musculoskeletal system/Neuromusculoskeletal and movement-related functions /structures related with movement

13. Diseases of the genitourinary system/Functions & structures of Genitourinary system

14. Pregnancy, childbirth/functions & structure of the reproductive system

15. Conditions originating in the perinatal period/Congenital malformations, deformations and chromosomal abnormalities

16. Injury, poisoning and other consequences of external causes/External causes of morbidity and mortality (accidents, self-harm, assault etc.)

17. Self-reported general health measures

18. Social outcomes (e.g. income, occupational mobility)

19. Multiple biomedical outcomes

20. Multiple biomedical and social outcomes

21. Mortality

22. Behavioural risk factors (e.g. physical activity, alcohol and cigarette use)

23. Other

^c^ Denominator (total number of classifiable papers) used to calculate %. Total % > 100% as some papers reported on >1 outcome and/or >1 exposure

^d^ Life stages defined as follows: prenatal; infancy (0-2); childhood (2-12); adolescence (13-19); adult (20-64); older adult (65+)

^e^ Setting defined according to World Bank classifications <http://data.worldbank.org/about/country-and-lending-groups>

**Supplementary table 2: Summary of findings from classification of abstracts identified by literature search 2 stratified by year of publication (i.e. Medline search for papers using the term ‘life course’ in their title and/or abstract (1990-2014), see text box for details of this search)**

|  | **%** | | | |
| --- | --- | --- | --- | --- |
|  | **1990-1996** | **1997-2002** | **2003-2008** | **2009-2014** |
| **Maximum N** | 89 | 211 | 664 | 1232 |
| **Type of paper** (N=2196^a^)  Empirical  Methods – statistical or study design  Conceptual, narrative or qualitative reviews  Systematic review  Policy  Other  Unclassifiable | 50.6  5.6  37.1  0  1.1  5.6  0 | 57.8  5.2  29.9  0.5  2.4  4.3  0 | 61.1  7.4  24.9  2.3  2.4  1.5  0.5 | 59.8  5.3  27.7  1.5  4.4  0.7  0.7 |
| **Outcomes^b^** (N=1694^c^)  1. Infectious and parasitic diseases  2. Cancer  3. Blood and blood-forming organs  4. Endocrine, nutritional and metabolic  5. Mental and behavioural  6. Nervous system  7. Eye, ear & related structures  8. Cardiovascular system  9. Respiratory system  10. Digestive system  11. Skin and related structures  12. Musculoskeletal system  13. Genitourinary system  14. Pregnancy, childbirth & the reproductive system  15. Conditions originating in the perinatal period  16. Injury, poisoning and other consequences of external causes  17. Self-reported general health measures  18. Social outcomes  19. Multiple biomedical outcomes  20. Multiple biomedical and social outcomes  21. Mortality  22. Behavioural risk factors  23. Other | 0  1.6  0  0  33.3  0  0  1.6  0  0  0  4.8  0  3.2  0  3.2  14.3  25.4  3.2  1.6  7.9  12.7  4.8 | 0  1.4  0  4.3  30.9  0  0  10.8  1.4  2.2  0  2.9  0  5.0  0  2.2  12.2  17.3  1.4  0.7  8.6  11.5  5.0 | 0.2  4.3  0.4  8.7  20.6  1.4  0.4  11.9  1.4  2.8  0  3.4  0.8  4.3  0.2  1.4  9.3  6.1  2.6  0.6  6.7  11.7  12.8 | 0.7  3.2  1.2  11.5  20.5  1.9  0.6  8.9  1.2  2.5  0.1  1.7  1.0  5.6  0.5  1.6  5.5  7.8  5.1  1.5  4.1  8.5  12.3 |
| **Exposures** (N=1477^c^)  Socioeconomic environment  Psychosocial environment (extrinsic)  Physical environment  Cognitive  Body size and shape and maturation  Emotional (intrinsic factors)  Behavioural risk factors  Genetics  Epigenetics  Other | 26.1  32.6  2.2  0  4.3  4.3  4.3  2.2  2.2  50.0 | 41.2  26.0  2.3  4.6  6.9  6.9  16.8  0  0  31.3 | 45.6  23.5  2.5  6.2  13.7  6.6  11.8  3.2  1.1  25.1 | 40.3  20.2  2.9  2.9  13.4  8.1  10.6  4.4  2.4  28.6 |
| **Timing of exposures** (N=1239)  Pre-adult (<20 y)  Adult (≥ 20 y)  Both pre-adult and adult  Intergenerational | 13.9  55.6  30.6  0 | 17.7  32.7  48.7  0.9 | 13.7  31.5  51.6  3.2 | 17.2  26.5  50.7  5.7 |
| **Type of analyses** (N=1135)  Associational study covering 1 life stage^d^  Associational study covering ≥ 2 life stages^d^  Repeated measures/trajectories modelled | 58.3  37.5  4.2 | 33.3  54.8  11.8 | 25.1  62.4  12.5 | 20.6  60.6  18.8 |
| **Setting^e^** (N=1230)  UK  North America (incl USA and Canada)  Mainland Europe  Other high income country  Low to middle income country | 9.1  69.7  18.2  3.0  0 | 23.2  44.6  17.0  11.6  3.6 | 25.7  43.1  16.8  7.2  7.2 | 18.3  44.3  18.7  7.0  11.7 |

^a, b, c, d, e^ See footnotes for supplementary table 1
