# Supplementary material for: The last two decades of life course epidemiology, and its relevance for research on ageing
Source: Int J Epidemiol. 2016 Oct 6;45(4):973–88. doi: 10.1093/ije/dyw096 (PMC5841628; doi:10.1093/ije/dyw096)
Supplement: Supplementary Data [file supp_45_4_973_v2_index.html]

Supplementary Data 

# The last two decades of life course epidemiology, and its relevance for research on ageing

## Supplementary Data

files

- Supplementary Data - docx file
